# Widespread pain in axial spondyloarthritis: clinical importance and gender differences

**DOI:** 10.1186/s13075-018-1626-8

**Published:** 2018-07-27

**Authors:** Thijs Willem Swinnen, René Westhovens, Wim Dankaerts, Kurt de Vlam

**Affiliations:** 1 Division of Rheumatology, University Hospitals Leuven, Herestraat 49, 3000 Leuven, Belgium; 2 Skeletal Biology and Engineering Research Center, Department of Development and Regeneration, KU Leuven, Herestraat 49 box 7003/13, 3000 Leuven, Belgium; 3 Musculoskeletal Rehabilitation Research Unit, Department of Rehabilitation Sciences, KU Leuven, Tervuursevest 101 box 1501, 3001 Leuven, Belgium

**Keywords:** Ankylosing spondylitis, Widespread pain, Anxiety, Disability, Inflammation, Depression, Body chart, Gender differences

## Abstract

**Background:**

There is a remarkable lack of detailed knowledge on pain areas in axial spondyloarthritis (axSpA), and their clinical relevance is largely unknown. Pain area may reflect local disease processes, but amplification of nervous system signalling may alter this relationship. Also, gender differences in pain area may exist in axSpA, possibly confounding disease activity outcomes. Therefore, we firstly detailed pain locations in axSpA and evaluated gender differences. Secondly, we explored the relationship of regional pain definitions with clinical outcomes. Finally, we explored the role of pain area in the assessment of disease activity.

**Methods:**

Body charts informed on the presence of axial, peripheral articular and non-articular pain in 170 patients (108 men, 62 women) with axSpA. Multivariate Odds Ratios (ORs) were used to compare genders. General linear models were used to explore clinical differences in disease activity (Bath Ankylosing Spondylitis Disease Activity Index [BASDAI]), activity limitations (Bath Ankylosing Spondylitis Functional Index [BASFI]), fear of movement (Tampa Scale for Kinesiophobia 11-item version [TSK-11]), anxiety (Hospital Anxiety and Depression Scale subscale anxiety [HADS-A]) and depression (HADS subscale depression [HADS-D]) between four subgroups classified by widespread non-articular pain (WNAP+/−) and physician global assessment of disease activity (PGDA+/−) (*p* < .05). Principal Component Analysis (PCA) was performed to explore gender differences in the structure of disease activity.

**Results:**

Axial thoracic pain was least prevalent (lumbar, 74.4%; cervical, 47.6%; cervicothoracic, 47.6%; thoracic, 32.4%), but it was about three times more likely in women (OR, 2.92; *p* = .009). Axial cervicothoracic junction pain spread more diffusely in women (OR, 2.48; *p* = .018). Women exhibited a two- to threefold increased likelihood of widespread axial (OR, 3.33; *p* = .007) and peripheral articular (OR, 2.34; *p* = .023) pain. A subgroup of WNAP+/PGDA− combined with low PGDA (27% of all patients) was associated with worse BASFI, BASDAI, HADS-A and HADS-D in men and worse TSK-11 and HADS-A in women (*p* < .05). Disease activity outcomes showed a two-factor structure in women but not in men.

**Conclusions:**

In patients with axSpA, the location and spread of pain was different between genders and was related to worse clinical status. On the basis of pain area and PGDA, clinical subgroups exhibiting a remarkably distinct health status were identified. Outcome instruments such as BASDAI should acknowledge gender differences to ensure structural validity.

**Electronic supplementary material:**

The online version of this article (10.1186/s13075-018-1626-8) contains supplementary material, which is available to authorized users.

## Background

Disease processes in axial spondyloarthritis (axSpA) involve tissue inflammation seen as enthesitis, synovitis and bone marrow oedema, as well as structural damage in the form of erosion, fat metaplasia/backfill and bone formation (sclerosis, ankylosis) [1, 2]. Spinal articular features typically occur at intervertebral corners and end-plates, zygapophyseal, and costovertebral and costotransverse joints, as well as at spinal ligament insertions [2, 3]. Asymmetrical mono- or oligoarthritis and enthesitis may add peripheral aspects to the predominant axial disease presentation [2]. Extra-articular features such as psoriasis, anterior uveitis or inflammatory bowel disease further illustrate the systemic nature of axSpA [2]. Although the exact aetiology of axSpA is largely unknown, a complex interplay between genetics (e.g., HLA-B27 [2]), biomechanical stress within the enthesis organ [4], gut bacterial dysbiosis [5] and several dysfunctional immune-competent cells (e.g., innate lymphoid group 3 cells [6]) may elicit auto-inflammation driven by key cytokines tumour necrosis factor (TNF)-α, interleukin (IL)-17 and IL-23 [7].

Cardinal clinical signs and symptoms of axSpA include inflammatory pain, stiffness and impaired mobility in the axial region and peripheral joints. To a large extent, these features are thought to reflect adaptive pain-motor mechanisms associated with inflammation and consequences of bone formation associated with the disease [8, 9]. To capture these local tissue processes, the Assessment in SpondyloArthritis international Society (ASAS) expert group endorsed classification and response (ASAS20, ASAS40, ASAS 4/5) criteria, as well as disease activity (Bath Ankylosing Spondylitis Disease Activity Index [BASDAI], Ankylosing Spondylitis Disease Activity Index [ASDAS]) and spinal mobility (Bath Ankylosing Spondylitis Metrology Index [BASMI]) scales in axSpA [2]. These mainly include self-reported numerical rating scales used to assess axial/peripheral pain intensity and spinal stiffness duration, but they also include clinical examination findings (e.g., joint effusion) and metrology using a tape measure [2].

Although useful for research, the mere focus on inflammation in the assessment of body structures and functions as proposed by ASAS may have pitfalls in clinical practice. Firstly, nociceptive/mechanical [10], neuropathic [11] or dysfunctional [12] pain mechanisms may complicate the clinical picture in axSpA and concurrently may influence pain intensity or stiffness duration scales. For example, ongoing low-grade or intense episodes of inflammation in axSpA likely induce a bottom-up amplification of neural signalling in the central nervous system that leads to pain hypersensitivity and to the spread of pain in a broader area, a process known as *central pain plasticity* (*central sensitization*) [13]. Also, psychological factors have been shown to exert top-down effects on disease activity estimates in axSpA [14]. Clinically, these pain mechanisms may translate to widespread pain, a feature seen in about 2–34% of patients with axSpA [12, 15, 16]. Secondly, ASAS outcome instruments including cut-offs for disease status or treatment response assume homogeneity in the axSpA population. Recently, gender differences in disease activity items such as higher reported pain intensity [17] or lower spinal mobility measures [18] have increasingly been observed in axSpA. Because cut-off levels might impact treatment decisions, this issue cannot be underestimated.

As a first step to improve the clinical assessment of pain in axSpA, the aim of this cross-sectional study was to explore the value of a more detailed pain area assessment as an adjunct to axial and peripheral articular pain intensity and stiffness. More specifically, the aims of this study were as follows:

1. To evaluate the prevalence of pain in anatomically distinct body regions and the body locations within these regions (topographical pain analysis)
2. To determine the association between the extent of axial, peripheral articular and peripheral non-articular pain areas and clinical variables (activity limitations, spinal mobility, disease activity, anxiety and depression)
3. To explore the role of assessing axial, peripheral articular and peripheral non-articular pain areas in the evaluation of the disease activity (using factor analysis)
4. To evaluate gender differences in all analyses.

## Methods

### Participants

Subjects (*n* = 190) with a definite diagnosis of axSpA according to the ASAS classification criteria [19], verified by an ASAS expert rheumatologist (KDV), were randomly included in this cross-sectional observational study. All patients were recruited from the outpatient spondyloarthritis clinic at the University Hospitals of Leuven, Belgium. Subjects with other inflammatory or systemic rheumatic conditions or who were unable to autonomously complete questionnaires in Dutch were excluded.

### Outcome measures

#### Anthropometrics and demographics

Height was measured with a stadiometer (Holtain Ltd., Dyfed, UK) to the nearest 0.1 cm, and weight was measured with a digital scale (SECA, Birmingham, UK) to the nearest 0.1 kg. Age (in yr), gender (male = 1/female = 2), disease duration (in yr), work status (yes = 1/no = 0) and the use of medication (biologicals, NSAIDs, DMARDs, analgesics, psychopharmacologics, corticoids, yes = 1/no = 2) were assessed during an interview and verified via the patient’s medical record.

#### Activity limitations

The ten-item BASFI numerical rating scale was used to assess patient-reported activity limitations [20]. The BASFI is an ASAS-endorsed instrument used to measure activity limitations with well-established psychometric properties in axSpA [2].

#### Spinal mobility

The BASMI was used to measure spinal mobility via five clinical tests, namely cervical rotation measured with a goniometer (accuracy 2 degrees; ORTEC Orthopedics, Leuven, Belgium) and a tape measure (accuracy 1 mm; Prym, Stolberg, Germany) of lumbar flexion, lumbar side flexion, tragus-to-wall distance and intermalleolar distance. For cervical rotation, lumbar side flexion and tragus-to-wall distance, the mean of the left and right measurements was taken, and all scores were converted according to the BASMI 10 scoring system [2]. The psychometric properties of the ASAS-endorsed BASMI in axSpA are well established [2, 21, 22].

#### Disease activity

The six-item BASDAI numerical rating scale was used to evaluate patient-reported disease activity. As recommended by ASAS, items 5 and 6 combined represented patient-reported inflammation [2]. The one-item Physician Global Assessment of Disease Activity (PGDA) numerical rating scale assessed during the routine rheumatology visit at inclusion represented physician-reported disease activity [2]. C-reactive protein (CRP, in mg/L) served as the laboratory-based disease activity marker [2]. The psychometric properties of the BASDAI, BASDAI inflammation, PGDA and CRP in axSpA are well established [2].

#### Fear of movement and (re)injury beliefs

The Tampa Scale for Kinesiophobia Dutch version with 11 items (TSK-11) was used to assess fearful beliefs regarding movement and (re)injury. Each item is provided with a 4-point Likert scale with scoring alternatives ranging from ‘strongly disagree’ to ‘strongly agree’ (range, 11–44). The psychometric properties of TSK-11 are well established in chronic pain populations [23] and recently in axSpA [24].

#### Anxiety and depression

The 14-item Hospital Anxiety and Depression Scale (HADS) was used to derive information on depression (7 items) and anxiety (7 items) using a 4-point Likert scale with higher values representing more depressed or anxious mood (range, 0–21 for each subscale). The psychometric properties of HADS are well established in axSpA [25].

#### Pain area

**Fig. 1:**
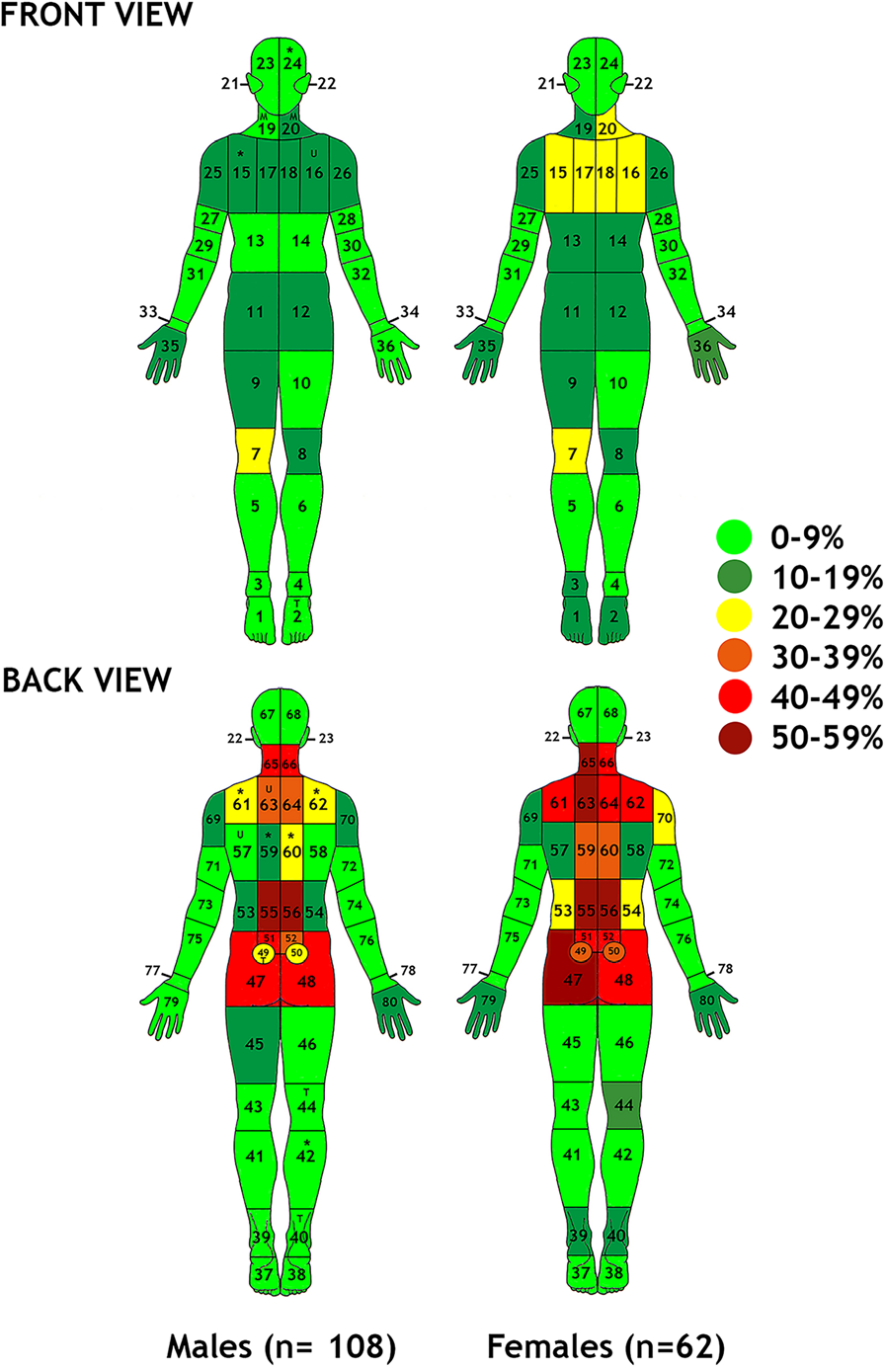
Graphical illustration of pain locations displayed as prevalence estimates for the total group and by gender in patients with axial spondyloarthritis (*n* = 170). * *p* < .05 in both univariate chi-square and multivariate logistical regression analyses; ^U^ *p* < .05 only in univariate analysis; ^M^ *p* < .05 only multivariate analysis; ^T^ *p* < .05 in univariate but trend in multivariate analysis

An anterior and posterior body chart filled in by the patient during the intake interview determined the presence of pain (yes = 1/no = 0) during the past week in 80 body locations (LOC) from which 22 body regions were derived post hoc (Fig. 1). Articular peripheral body locations were modified from the 76/74-joint count in psoriatic arthritis [26]. Non-articular peripheral body locations were taken from the widespread pain index as applied in the preliminary diagnostic criteria for fibromyalgia [27]. Axial body locations and regions were defined according to the International Association for the Study of Pain [28] and known axial pain referral patterns [29]. A detailed numeric description of pain locations within different body regions is given in Table 2. Widespread axial pain was defined as pain present in the lumbar (LX), thoracic (TX), cervicothoracic junction (CTJ) and cervical (CX) body regions. Widespread peripheral articular (WAP) and non-articular (WNAP) pain was considered present if the sum of painful (non-)articular peripheral body locations exceeded the median for this variable. Axial, peripheral articular and non-articular pain were also calculated as the sum of their respective body locations and expressed as percentages. Two blinded raters with a bachelor’s-level degree in physical therapy independently scored the body charts showing good to excellent reliability of this procedure (kappa range, 0.742–1.00; percentage of agreement range, 94–100%) (Additional file 1: Table S1).

### Data reduction and statistical analysis

Sample characteristics were presented as mean ± SD and median with IQR or frequencies as percentage. Normal distribution of all variables was evaluated with the Shapiro-Wilk test (*p* < .05). For each body region and location, prevalence with the corresponding 95% CI was calculated in the total group and for men and women separately. Cohen’s kappa values and percentage of agreement were calculated as inter-rater reliability coefficients (*n* = 170), and thresholds for further use of body charts were set at minimally good (0.61–0.80), preferably excellent (> 0.80) [30].

Pain area between genders was univariate compared with an independent *t* test for continuous variables or chi-square test for frequencies (*p* < .05). In case of low cell counts (fewer than five) for the latter, the chi-square test was replaced by the phi coefficient to obtain valid *p* values. For each body region and location as dependent variables, BASDAI inflammation, gender, BASMI, disease duration, age and PGDA were entered as independent variables in a multivariate logistic regression model with ORs and their 95% CIs as output for the gender comparison. The same independent variables were entered into a multiple linear regression model for axial, articular and non-articular pain sum scores with (un)standardized beta (b, stß) values and their 95% CIs reported for gender effects.

Pearson product-moment correlation coefficients determined the univariate association between axial, peripheral non-articular and articular pain versus clinical variables (*p* < .05). Further, clinically relevant subgroups based on the median split of physician-reported disease activity and peripheral non-articular pain (PGDA−/WNAP−, PGDA−/WNAP+, PGDA+/WNAP−, PGDA+/WNAP+) were compared in a multivariate general linear model with subgroup and gender as between-subject factors; BASMI, BMI, disease duration and age as covariates; and BASDAI, BASFI, BASMI, HADS depression and HADS anxiety separately as dependent variables (*p* < .05). Results of post hoc tests were presented with uncorrected (*p* < .05) and Bonferroni-corrected *p* values.

PCA was performed to explore the contribution of all six items of BASDAI and axial, peripheral non-articular and articular pain sum scores in assessing disease activity. The covariance structure was analysed, and variables with an eigenvalue ≥ 1 were retained. Also, the scree plot was visually inspected to confirm the number of extracted factors. Factor loadings were varimax-rotated, and the variance explained per factor was reported. The magnitude of the rescaled rotated factor loadings determined factor membership for each variable and a large > 0.60 or smaller < 0.60 contribution to the total variance explained (Fig. 3). On the basis of this membership, the factor axial disease activity and peripheral disease activity were calculated as the arrhythmic mean of all items within the developed factor without weighting. PCA was repeated per gender to explore structural variability in disease activity outcomes. All analyses were performed with IBM SPSS Statistics version 20.0 software (IBM, Armonk, NY, USA).

## Results

### Demographic and anthropometrical data

Of all 190 patients invited, only 4 refused to participate, resulting in 186 included patients. The numbers of cases with missing data were 2 (1.1%) for the BASMI, 3 (1.6%) for the TSK-11, 3 (1.6%) for the HADS, 7 (3.8%) for the BASFI, 6 (3.2%) for the BASDAI, 26 for PGDA (14.0%), 31 for CRP (16.7%) and 9 for work status (4.8%). Full data across outcome measures were available for 170 patients (9.1% data loss) for analyses without PGDA and CRP. Analyses considering PGDA and CRP included 133 subjects (28.4% data loss). No statistically significant differences were found between groups with or without missing data (*p* > .05). Descriptive statistics for the total sample and per gender are given in Table 1.

**Table 1:** Descriptive statistics for all demographic, anthropometric and disease-related outcomes in patients with axial spondyloarthritis (*n* = 170)

Variables	Total group (*n* = 170)		Men (*n* = 108)		Women (*n* = 62)		
	Mean (SD)	Med (IQR)	Mean (SD)	Med (IQR)	Mean (SD)	Med (IQR)	*p* Value
Age, yr	42.9 (12.2)	42.7 (20.3)	43.8 (12.5)	43.2 (20.4)	41.3 (11.5)	42.1 (18.0)	.199
Disease duration, yr	13.1 (11.1)	10.7 (16.6)	14.0 (11.2)	11.9 (18.4)	11.5 (11.0)	8.8 (13.0)	.155
Height, cm	171.6 (9.4)	172.3 (12.9)	176.2 (7.1)	176.2 (9.1)	163.7 (7.4)	163.4 (9.5)	*< .00*
Weight, kg	77.0 (15.0)	76.6 (20.9)	81.7 (13.9)	80.5 (19.2)	69.7 (13.4)	67.0 (18.1)	*< .00*
BMI, kg/m^2^	26.1 (4.4)	25.6 (6.4)	26.3 (4.4)	25.5 (6.1)	25.7 (4.4)	25.7 (7.1)	.408
BASDAI (0–10)	3.8 (2.1)	3.7 (3.3)	3.6 (2.2)	3.5 (3.5)	4.3 (2.0)	4.2 (3.1)	*.027*
PGDA (0–10) (*n* = 146)	1.4 (1.8)	1.0 (2.0)	1.4 (1.9)	0.9 (2.0)	1.4 (1.6)	1.0 (1.6)	.583
CRP, mg/L (*n* = 141)	8.3 (16.0)	2.9 (6.6)	8.9 (16.5)	3.0 (6.6)	7.4 (15.3)	2.2 (6.7)	.810
BASFI (0–10)	3.6 (2.4)	3.4 (3.8)	3.5 (2.4)	3.2 (4.0)	3.8 (2.3)	3.7 (3.7)	.459
BASMI (0–10)	3.0 (1.8)	2.8 (2.0)	3.3 (2.0)	2.8 (2.8)	2.6 (1.2)	2.6 (1.6)	*.002*
Cervical rotation, degrees	60.5 (19.6)	65.0 (25.0)	58.0 (21.3)	62.5 (27.8)	65.0 (15.6)	66.0 (20.5)	*.015*
Tragus to wall, cm	13.6 (4.6)	11.7 (4.5)	14.9 (5.0)	13.3 (6.2)	11.4 (2.3)	10.8 (1.8)	*< .00*
Lateral flexion, cm	12.4 (5.1)	12.7 (8.1)	11.7 (6.1)	12.1 (9.4)	13.5 (4.2)	13.1 (6.1)	*.028*
Intermalleolar distance, cm	99.0 (22.6)	103.3 (25.1)	101.0 (21.4)	105.2 (25.2)	95.5 (24.2)	100.7 (25.0)	.141
Modified Schober, cm	5.3 (2.1)	5.5 (2.5)	4.9 (2.3)	5.2 (3.1)	5.9 (1.7)	6.2 (2.0)	*.002*
TSK-11 (11–44)	24.8 (6.3)	25.0 (10.0)	27.8 (6.4)	25.0 (10.0)	24.9 (6.0)	25.0 (9.3)	.888
HADS depression (0–21)	4.6 (3.6)	4.0 (5.0)	4.8 (3.7)	4.0 (5.0)	4.4 (3.5)	3.0 (4.0)	.580
HADS anxiety (0–21)	7.1 (3.6)	7.0 (5.0)	6.7 (3.4)	7.0 (5.0)	7.8 (3.9)	7.5 (5.0)	.071
Frequencies (%)							
Gender, male/female	108/62 (64/36)		NA		NA		NA
NSAIDs, yes/no	87/83 (51/49)		56/52 (52/48)		31/31 (50/50)		.816
Biologicals, yes/no	67/103 (39/61)		45/63 (42/58)		22/40 (36/64)		.427
Corticosteroids, yes/no	12/158 (7/93)		6/102 (6/94)		6/56 (10/90)		.313^a^
DMARDs, yes/no	71/99 (42/58)		43/65 (40/60)		28/34 (45/55)		.496
Psychopharmacologic agents, yes/no	12/158 (7/93)		6/102 (6/94)		6/56 (10/90)		.313^a^
Analgesics, yes/no	73/97 (43/57)		33/75 (31/69)		40/22 (65/35)		*< .00*
Work status, yes/no^b^	99/64 (61/39)		64/38 (63/37)		35/26 (57/43)		.497

*Abbreviations: BMI* Body mass index, BASDAI Bath Ankylosing Spondylitis Disease Activity Index, *BASFI* Bath Ankylosing Spondylitis Functional Index, *BASMI* Bath Ankylosing Spondylitis Metrology Index, *CRP* C-reactive protein, normal value < 5 mg/L, *HADS* Hospital Anxiety and Depression Scale, *NSAIDs* Non-steroidal anti-inflammatory drugs, *DMARDs* Disease-modifying anti-rheumatic drugs, *p<.05*

^a^*p* Value based on phi coefficient instead of chi Square test

^b^*n* = 163 (9 males, 2 females missing)

### Prevalence and gender differences for pain regions

Full prevalence data for all pain regions for the total group and per gender are presented in Table 2. Left (10.0% [9.7–10.3%]) and right (11.2% [10.8–11.6%]) whole-leg pain was a rare finding in this axSpA group for both men and women. In contrast, pain in the lumbar spine (LX) was highly prevalent (total group, 74.4% [74.2–75.2%]) and significantly more prevalent in women (83.9% [83.5–84.3%]) than in men (69.4% [68.9–69.9%]) in univariate analysis only (chi-square test, 4.338, *p* = .037; OR, 1.74 [0.73–4.14], *p* = .210). Pain in the thoracic spine (TX) was remarkably less prevalent overall (32.4% [31.9–32.9%]) and in men (25% [24.5–25.5%]), but about three times more likely in women (45.2% [44.6–45.8%], chi-square test, 7.315, *p* = .007; OR, 2.92 [1.30–6.55], *p* = .009). Cervicothoracic junction (CTJ) pain was moderately prevalent overall (47.6% [47–48.2%]) and in men (48.1% [47.5–48.7%]), but about two and one-half times more likely in women (66.1% [65.6–66.6%], chi-square test, 5.139, *p* = .023; OR, 2.48 [1.17–5.26], *p* = .018). Pain in the cervical spine and head (CX and head) was also common overall (47.6% [47–48.2%]), but not significantly different between men (45.4% [44.8–46.0%]) and women (51.6% [51–52.2%]) (*p* > .05). Isolated occurrence of lumbar (LX only, 21.2% [20.7–21.7%]) and cervical (CX only, 10.6% [10.2–11.0%]) spine pain was common, but not in the thoracic spine (TX only, 2.4 [2.2–2.6%]). Also, no apparent gender differences existed (*p* > .05). Widespread axial pain was moderately prevalent (26.5% [26.0–27.0%]) and about three times more likely in women (38.7% [38.1–39.3%]) than in men (19.4% [18.9–19.9%]) (chi-square test, 7.511, *p* = .006; OR, 3.33 [1.38–8.02], *p* = .007). Widespread articular peripheral pain also showed a twofold increased likelihood in women (56.5% [55.9–57.1%]) compared with men (40.7% [40.1–41.3%]; chi-square test, 3.908, *p* = .048; OR, 2.34 [1.12–4.88], *p* = .023), whereas for widespread non-articular peripheral pain, statistical significance was not met (*p* = .079). The sum score for axial pain locations (total group mean ± SD, 36.1 ± 21.1; median [IQR], 27.3 [28.4]) was significantly higher in women (mean ± SD, 37.5 ± 20.8; median [IQR], 36.4 [36.4]) than in men (mean ± SD, 28.3 ± 20.7; median [IQR], 27.3 [31.9]; t-test *p* = .006; b = 8.52 [1.42–15.63]; stß = .193; *p* = .019). No gender differences were found for articular (total group mean ± SD, 9.2 ± 12.3; median [IQR], 3.8 [15.4]) and non-articular (total group mean ± SD, 7.6 ± 10.0; median [IQR], 4.2 [12.5]) peripheral pain sum scores (*p* > .05).

**Table 2:** Prevalence estimates and gender differences in painful body regions in patients with axial spondyloarthritis (*n* = 170)

Body region	Location numbers	Total group	Males (*n* = 108)	Females (*n* = 62)	Chi-square value	*p* Value	OR^a^	*p* Value
Leg right	41, 43, 45, 47	11.2 (10.8–11.6)	10.2 (9.9–10.5)	12.9 (12.5–13.3)	0.293	.588	1.35 (0.43–4.30)	.588
Leg left	42, 44, 46, 48	10 (9.7–10.3)	9.3 (9–9.6)	11.3 (10.9–11.7)	0.181	.671	0.77 (0.21–2.77)	.687
SIJ	49, 50	32.9 (32.4–33.4)	26.9 (26.4–27.4)	43.5 (42.9–44.1)	4.971	**.026**	1.76 (0.82–3.81)	.149
LX	47–56	74.7 (74.2–75.2)	69.4 (68.9–69.9)	83.9 (83.5–84.3)	4.338	**.037**	1.74 (0.73–4.14)	.210
TX	57–60	32.4 (31.9–32.9)	25 (24.5–25.5)	45.2 (44.6–45.8)	7.315	**.007**	2.92 (1.30–6.55)	**.009**
CTJ	61–64	47.6 (47–48.2)	48.1 (47.5–48.7)	66.1 (65.6–66.6)	5.139	**.023**	2.48 (1.17–5.26)	**.018**
CX	65, 66	47.6 (47–48.2)	45.4 (44.8–46.0)	51.6 (51–52.2)	0.615	.433	1.55 (0.75–3.23)	.240
CX and head	19–24, 65–68	54.7 (54.1–55.3)	50.9 (50.3–51.5)	61.3 (60.7–61.9)	1.708	.191	1.71 (0.81–3.60)	.159
Sternum	17, 18	20.6 (20.1–21.1)	18.5 (18.1–18.9)	24.2 (23.7–24.7)	0.776	.378	0.98 (0.39–2.48)	.972
LX only	47–56	21.2 (20.7–21.7)	20.4 (19.9–20.9)	22.6 (22.1–23.1)	0.115	.734	0.97 (0.41–2.30)	.944
TX only	57–60	0.6 (1.5–0.7)	0.0 (0.0–0.0)	1.6 (1.5–1.7)	0.102^b^	.186	0.00 (0.00–0.00)	.999
CX only	61–68	10.6 (10.2–11.0)	12.0 (11.5–12.4)	8.1 (7.8–8.4)	0.657	.418	1.02 (0.31–3.34)	.999
Widespread axial pain	47–66^c^	26.5 (26.0–27.0)	19.4 (18.9–19.9)	38.7 (38.1–39.3)	7.511	**.006**	3.33 (1.38–8.02)	**.007**
Widespread peripheral articular pain	1–4, 7, 8, 25, 26, 29, 30, 33–36, 37–40, 43, 44, 73, 74, 77–80	46.5 (45.9–47.1)	40.7 (40.1–41.3)	56.5 (55.9–57.1)	3.908	**.048**	2.34 (1.12–4.88)	**.023**
Widespread peripheral non-articular pain	5, 6, 9–14, 25–28, 31, 32, 41, 42, 45, 46, 69–72, 75, 76	44.7 (44.1–45.3)	39.8 (39.2–40.4)	53.2 (52.6–53.8)	2.866	.090	1.97 (0.93–4.15)	.079

*Abbreviations: SIJ* Sacroiliac joint, *LX* Lumbar spine, *CX* Cervical spine, *TX* Thoracic spine, *CTJ* Cervicothoracic junction

^a^Multivariate OR ± 95% CI based on logistic regression analysis correcting for age, disease duration, spinal mobility (Bath Ankylosing Spondylitis Metrology Index), disease activity (Bath Ankylosing Spondylitis Disease Activity Index and physician global assessment of disease activity (*n* = 146)

^b^Phi value is given because of low cell frequency (fewer than cases); significant results in bold; *p* < .05

^c^ Positive if pain is present in regions 47–56 and 57–60 and in regions 61–64 and 65–66

### Within-region prevalence and gender differences in pain locations

Full prevalence data for all 80 pain locations for the total group and per gender are presented in Additional file 2: Table S2 and graphically summarized in Fig. 1. Overall, the dominant axial involvement in axSpA was clearly confirmed for both genders. Of note was a higher prevalence of pain in the anterior right knee (LOC 7, 21.8% [21.3–22.3%]), regardless of gender. In addition to the predominance of regional CX and CTJ pain in women, the within-region results also confirmed the increased lateral spread of pain in the CTJ region in women (LOC 61, 45.2% [44.6–45.8%]; LOC 62, 41.9% [41.3–42.5%]) compared with men (LOC 61, 26.9% [26.4–27.4%]; chi-square test, 5.925, *p* = .015; OR, 2.23 [1.05–4.71], *p* = .036; LOC 62, 24.1% [23.6–24.6%]; chi-square test, 5.918, *p* = .015; OR, 2.48 [1.14–5.42], *p* = .023). A similar effect did not reach significance in the thoracic or lumbar region (*p* > .05).

### Relationship of pain regions with disease-related outcomes

**Fig. 2:**
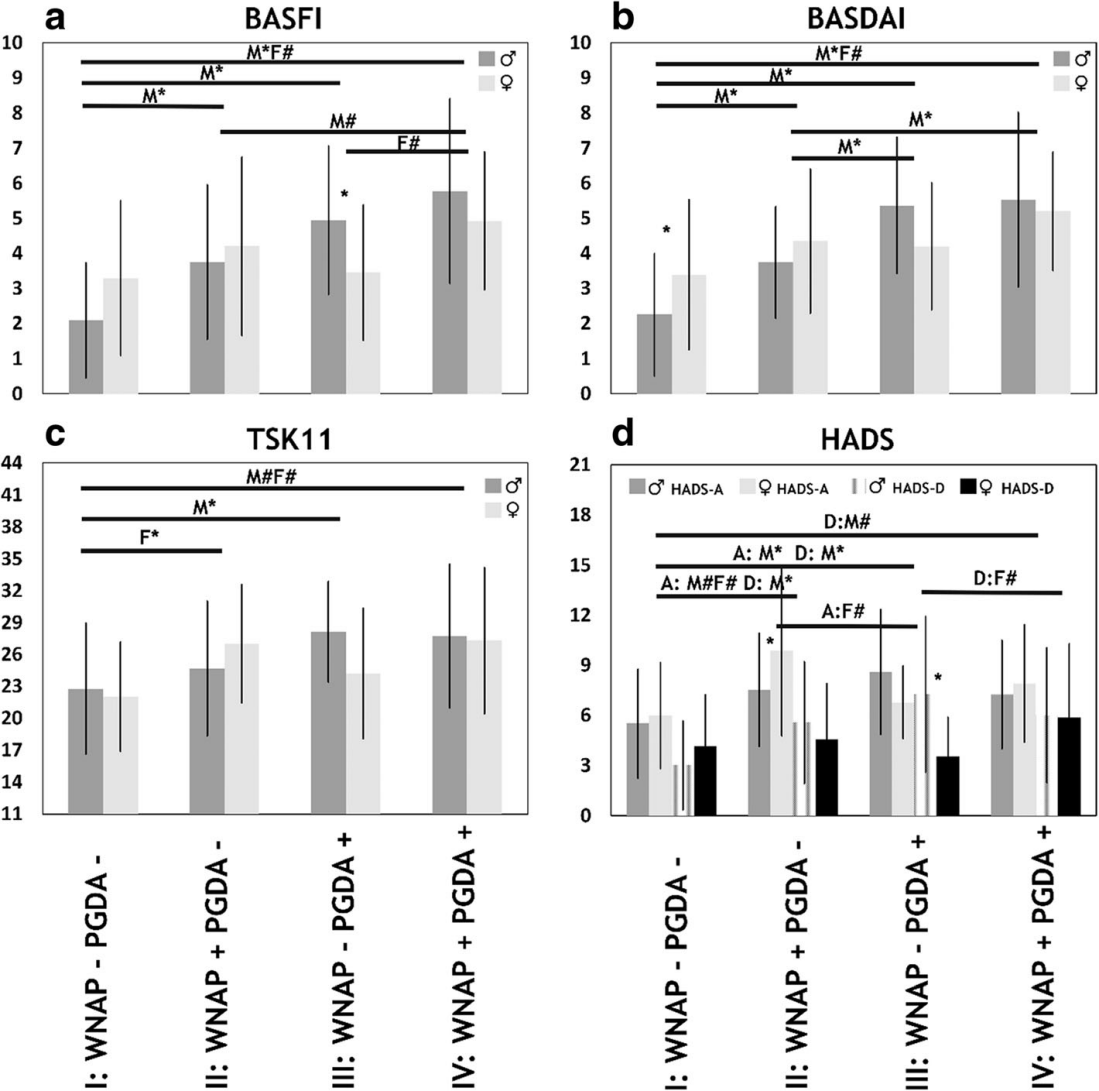
Clinical subgroups based on the presence or absence of widespread non-articular pain (WNAP) and physician global assessment of disease activity (PGDA) and their relationship with clinical variables (panel **a**-**d**) in axial spondyloarthritis (*n* = 146). *BASFI* Bath Ankylosing Spondylitis Functional Index (0–10), *BASDAI* Bath Ankylosing Spondylitis Disease Activity Index (0–10), *TSK11* 11-item version of Tampa Scale for Kinesiophobia (11–44), *HADS-D/A* Hospital Anxiety and Depression Scale (HADS) subscale anxiety (A) or depression (D) (0–21), ♂/♀ and *M/F* Male/female, *n* (%) group I: 59 (40), group II: 40 (27), group III: 24 (16), group IV: 23 (16). *^,#^ *p* < .05 indicating significant Bonferroni and uncorrected general linear model results between and within groups per gender, respectively

Univariate correlations between all variables for the total group and per gender can be found in Additional file 3: Table S3. General linear models (Fig. 2, Additional file 4: Table S4) revealed a graded relationship between subgroups combining non-articular peripheral pain and PGDA with BASFI, BASDAI, TSK-11 and HADS in men (*p* < .05). Of clinical interest was the subgroup of non-articular peripheral pain combined with low PGDA (WNAP+/PGDA−; 27% of all patients) that was associated with worse BASFI, BASDAI, and HADS anxiety and depression in men and worse TSK-11 and HADS anxiety in women (*p* < .05).

### Role of pain area in disease activity

**Fig. 3:**
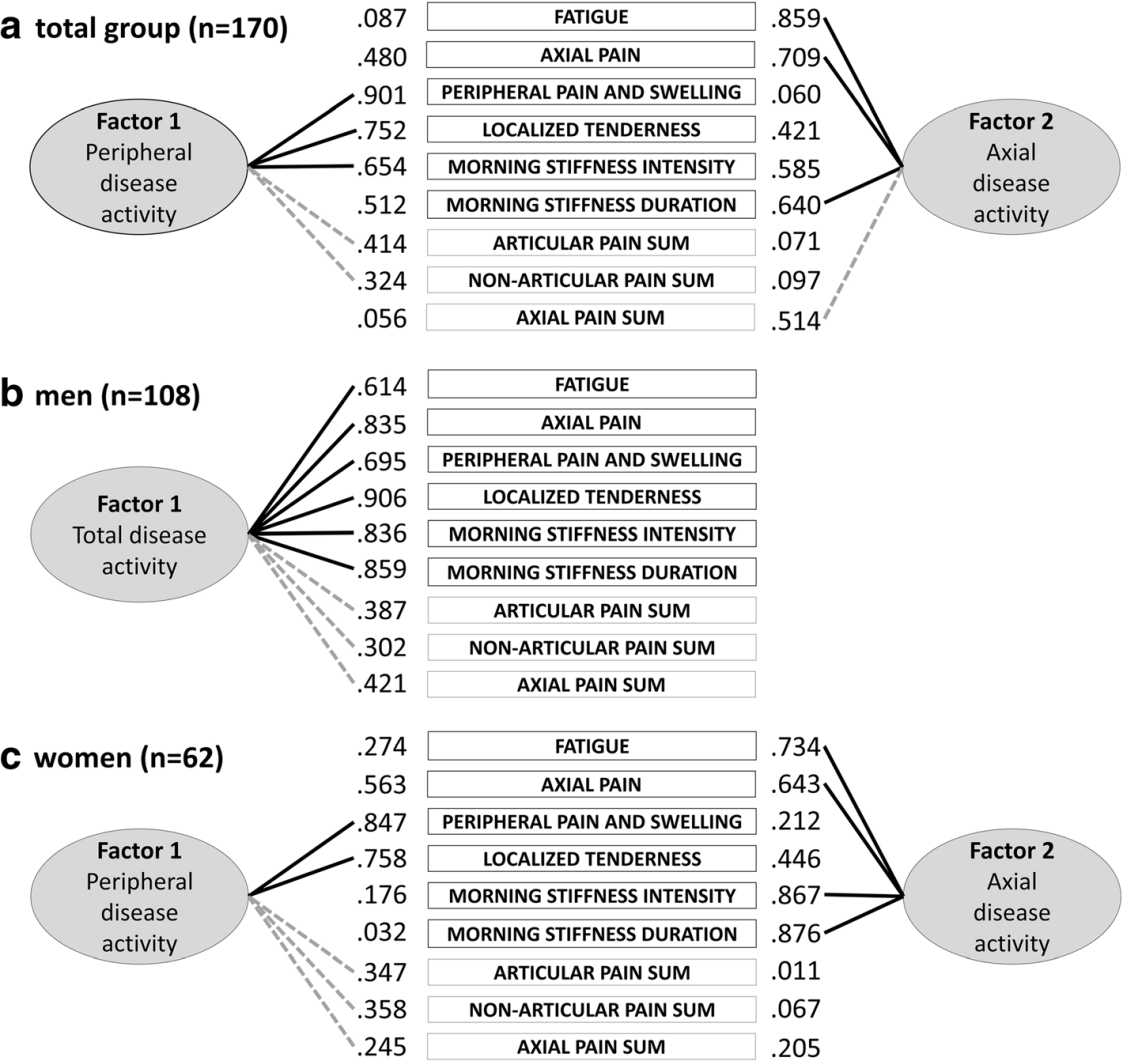
Graphical illustration of the contribution of each Bath Ankylosing Spondylitis Disease Activity Index item and spinal, articular and non-articular pain sum scores to peripheral and axial disease activity factors* in men and women with axial spondyloarthritis (*n* = 170). BASDAI, Bath Ankylosing Spondylitis Disease Activity Index; (non)-articular and spinal pain sum of pain area estimates were based on body charts (0–100%). * PCA with varimax rotation in the total group (**a**), men (**b**) and women (**c**). Rescaled rotated factor loadings are presented. Level of statistical significance was set at *p* < .05. Lines connect items to the construct they represent. *Solid* or *dashed lines* represent larger (> .60) or smaller (< .60) contributions to the underlying construct, respectively

Factor analysis revealed a differential role for body regions in the assessment of disease activity (Fig. 3). Axial pain sum score loaded on an axial disease factor together with BASDAI items 1, 2 and 6 to explain 11% of the variance. Non-articular and articular peripheral pain sum score loaded on a peripheral disease activity factor explained 56% of the variance. Importantly, the factor structure did vary between genders with a single- and two-factor solution in men and women, respectively.

## Discussion

To the best of our knowledge, this was the first study to detail the topography of pain in axSpA and to relate these findings to key clinical outcomes and the structural properties of BASDAI, the most commonly used self-reported method to assess disease activity. In the first part of the study, the prevalence of pain in clinically meaningful body regions was analysed. The observed dominant axial pain involvement likely confirmed axial disease, as enforced by the ASAS axSpA inclusion criteria [19]. The prevalence of pain was highest in the LX region (75%) when compared with the TX (34%), CTJ (48%) and CX (48%) regions. Indeed, the LX region was reported to be the first (LX: 67%, buttock: 40%, TX: 23.3%, CX/CTJ: 11.1%) and dominant (LX, 90%; buttock, 75%; TX, 55%) area affected in a large recent-onset inflammatory back pain cohort [31]. Comparable descriptions of axial pain locations in historic axSpA cohorts do not exist [32]; however, preferential thoracic inflammation and bone formation (thus discrepant with our dominant LX pain symptoms) have been reported [33]. We also found a strikingly low prevalence of unique pain involvement of the LX (20%), TX (1%) and CX (10%) regions. Blachier et al. [34] recently reported a similarly low TX (2%) but contrasting low LX (2%) with high SIJ (25%) prevalence of single inflammatory lesions visualised by magnetic resonance imaging (MRI). The mismatch at the lumbar level in our study can be explained by referral of pain in the LX region owing to SIJ inflammation [29, 35] or common local LX pain with a non-inflammatory origin, a differentiation that needs further study. For the peripheral joints, the more pronounced involvement of the right anterior knee may reflect increased loading of the dominant limb [36] and fits with the proposed link between biomechanics and disease processes in axSpA [37].

In the second part of the study, gender comparisons in regional pain prevalence revealed increased pain in the TX and CTJ, but not the CX region, in women. In the absence of other research on this topic, the observed gender differences in thoracic pain represent a novel finding. In contrast, the results of our study for the CTJ region probably coincided with the marked but ill-defined CX involvement in women that has been reported previously in both radiographic [32] and early axSpA cohorts [38]. Interestingly, and unique to this study, further within-region analysis revealed that the lateral spread of CTJ and sternal pain is more prevalent in women. On the basis of previous work on anterior chest pain, researchers have reported rather similar sternal pain occurrence between men and women, but focused on local joint pain only [39, 40].

In the third part of the study, on the widespread nature of pain and its clinical correlates, this cohort showed a fairly high occurrence of widespread spinal pain (27%) and spinal pain sum scores (35%), regardless of disease status and being even more pronounced in women (39% and 38%, respectively). Although rooted in different criteria definitions, these numbers mimic the prevalence of fibromyalgia in women with axSpA (39% versus range 11–34%) typically reported in the literature, but they were higher than expected in men (19% versus range 2–9%) [12, 15, 16, 27]. The latter may indicate incomplete correction for pain caused by partial bone formation via BASMI, a process known to affect the spine more in men [17]. These findings need careful interpretation, however, because multiple mechanisms may lead to a wider spread of spinal pain. Commonly involved anterior (e.g., anterior vertebral corner) versus posterior (e.g., zygapophyseal joint) spinal structures in axSpA have been shown to exhibit multisegmental/bilateral to unisegmental/unilateral innervation, respectively [41]. Consequently, the amount of disease processes, as well as the innervation pattern of the specific local tissues involved, likely results in a variable pain extent via local mechanisms of nociceptor activation and peripheral sensitization (primary hyperalgesia) [29, 35]. Also, central neural plasticity likely augments pain area and intensity in axSpA, especially in women [42], owing to activity- and transcription-dependent long-term potentiation (mono- and heterosynaptic) recruiting nearby receptive fields, a changed neural membrane excitability, the disinhibition of anti-nociceptive and facilitation of pro-nociceptive top-down pathways, all strongly influenced by immune-competent cells such as microglia (secondary hyperalgesia and widespread pain) [13].

To date, researchers in the few preliminary studies on pain physiology in axSpA have reported normal [43] (also in response to anti-TNF treatment [44]) to even elevated [45] pain pressure thresholds compared with control subjects and a moderate relationship with depression [46], non-superiority of algometry over manual palpation in the evaluation of entheseal pain [47] and pain-related brain morphology changes in response to anti-TNF treatment [11, 44], but all studies lacked proper gender comparisons. (For a recent mechanistic overview of gender differences in clinical and experimental non-axSpA pain research, *see* [42].) The observed increased widespread articular pain and trend for non-articular pain in women have been reported inconsistently in the literature [17, 32] and require a conclusive differentiation of local disease versus pain mechanisms. Further characterisation of the subgroup ‘low disease activity but high spread of pain’ (PGDA−/WPIP+) in this study, exhibiting a large burden of disease and association with anxiety in both genders and with depression in males, may help in this respect. Future work should concurrently include state-of-the-art assessment of inflammation/bone formation, pain mechanisms (including fibromyalgia criteria), psychosocial variables and clinical outcomes to unravel the spread of pain by gender, especially in clinically relevant subgroups of axSpA.

In the last part of the study, we revealed a two-factor structure of BASDAI that was linked to female gender and suggests the importance of considering axial and peripheral disease activity separately in women. Only one study reported a one-factor structure of BASDAI in both ankylosing spondylitis (*n* = 211, 82% men) and early spondyloarthritis (*n* = 86, 56% men), results that likely diverge from those of our study owing to lesser representation of women and small sample size [48]. Screening existing disease activity instruments in axSpA for gender compatibility is urgently needed. The impact of gender on cut-offs to define disease activity using the recently developed ASDAS in women has been reported [49] and may add to explanation of the lower response to biological therapy in women [50].

This study has a few limitations. First, we corrected all pain area estimates for differences in clinical outcome but were not able to include the ASDAS (missing patient global assessment) or MRI (feasibility and cost) to study local inflammation. In the absence of a gold standard [2], it is a strength, however, that objective and valid surrogate measures PGDA and CRP were included [51]. Similarly, plain radiographs were not available to evaluate bone formation, which was reflected by the reliable and valid BASMI [21]. Second, although this is the largest study reported to date on detailed topographical pain analysis axSpA, it must be recognized that our subgroup analysis is (for sample size reasons) exploratory. As a consequence, relevant differences between subgroups may have been missed. To tackle this power issue, we presented the uncorrected and corrected *p* values.

## Conclusions

This study describes the topography of pain in axSpA in detail. Apart from the dominant axial prevalence of pain, especially in the LX region, women more frequently exhibited TX and CTJ pain with a wider lateral spread, regardless of disease status. Our finding of widespread (non-articular) peripheral pain in combination with low PGDA questions current clinical decision-making using disease activity measures in a subgroup of axSpA. Also, the two-factor structure of disease activity found in women should be considered in the development of outcome instruments in axSpA.

## Additional files

Additional file 1: **Table S1.** Inter-rater reliability for each body chart region for the topographical pain assessment in patients with axial spondyloarthritis (*n* = 170). Reliability data for each body chart location. (XLSX 18 kb)

Additional file 2: **Table S2.** Painful body locations expressed as prevalence estimates for the total group and by gender in patients with axial spondyloarthritis (*n* = 170). Detailed analysis of prevalence outcomes per body location and per gender. (XLSX 19 kb)

Additional file 3: **Table S3.** Univariate Pearson product-moment correlations between all pain area estimates and clinical outcomes in patients with axial spondyloarthritis (*n* = 170). Correlation table for all pain area estimates and clinical outcomes. (XLSX 19 kb)

Additional file 4: **Table S4.** General linear model results comparing clinical outcomes between subgroups based on WNAP and PGDA status across and within genders in patients with axial spondyloarthritis (*n* = 146). Full subgroup comparison data across and within genders. (XLSX 15 kb)

## Abbreviations

+/−: Is/is not present
ASAS: Assessment in SpondyloArthritis international Society
ASDAS: Ankylosing Spondylitis Disease Activity Index
axSpA: Axial spondyloarthritis
β: Standardized β-value
b: Unstandardized β-value
BASDAI: Bath Ankylosing Spondylitis Disease Activity Index
BASFI: Bath Ankylosing Spondylitis Functional Index
BASMI: Bath Ankylosing Spondylitis Metrology Index
CRP: C-reactive protein
CTJ: Cervicothoracic junction body region
CX: Cervical body region
DMARDs: Disease-modifying anti-rheumatic drugs
HADS: Hospital Anxiety and Depression Scale
HLA: Human leucocyte antigen
IL: Interleukin
LOC: Body location(s)
LX: Lumbar body region
MRI: Magnetic resonance imaging
NSAIDs: Non-steroidal anti-inflammatory drugs
ORs: Odds Ratios
PCA: Principal Component Analysis
PGDA: Physician global assessment of disease activity
SIJ: Sacroiliac joint region
TNF: Tumour necrosis factor
TSK-11: Tampa Scale for Kinesiophobia 11-item version
TX: Thoracic body region
WAP: Widespread peripheral articular
WNAP: Widespread non-articular pain
